# Can pelvic tilt be restored by spinal osteotomy in ankylosing spondylitis patients with thoracolumbar kyphosis? A minimum follow-up of 2 years

**DOI:** 10.1186/s13018-018-0874-2

**Published:** 2018-07-09

**Authors:** Tianhao Wang, Yongfei Zhao, Guoquan Zheng, Yao Wang, Chunguo Wang, Zheng Wang, Yan Wang

**Affiliations:** 1 Southwest Hospital, Third Military Medical University, Chongqing, 400038 China; 2 Department of Orthopaedics, General Hospital of People’s Liberation Army, Beijing, 100853 China

**Keywords:** Ankylosing spondylitis, Osteotomy, Pelvic tilt, Sagittal balance

## Abstract

**Background:**

Defining the postoperative pelvic tilt (PT) individually can help to reconstruct sagittal balance. However, the postoperative actual PT is hardly restored to theoretical value. Some cases with theoretical postoperative PT was overcorrected and still did not have normal horizontal visual field after surgery. The objective of this study is to describe the pelvic tilt change after spinal osteotomy in ankylosing spondylitis (AS) kyphotic deformity and evaluate the effect on clinical outcomes.

**Methods:**

Twenty-three AS patients including 21 men and two women with thoracolumbar kyphosis, who underwent spinal osteotomy from 2013 to 2015 in our center, were retrospectively reviewed. A series of parameters including sacral slop (SS), pelvic incidence (PI), PT, and sagittal vertical axis (SVA) measured on preoperative and postoperative standing radiographs were analyzed. The theoretical postoperative PT (tPT) was calculated by the formula tPT = 0.37 × PI − 7. The radiographic measurements were compared before surgery, 2 weeks and at least 2 years postoperatively. Clinical outcomes were performed with the Oswestry disability index and Scoliosis Research Society-22 surveys.

**Results:**

Mean age of the patients (2 women, 21 men) was 39.8 ± 9.1 years. Mean follow-up was 27.4 ± 3.8 months, at least 24 months. After spinal osteotomy, SS and SVA were corrected from 11.9° ± 11.2° and 18.0 ± 7.6 mm preoperatively to 25.8° ± 8.1° and 9.6 ± 6.3 mm postoperatively, respectively (*p* < 0.001). PT reduced from 37.6° ± 12.1° to 21.8° ± 9.8° postoperatively (*p* < 0.001). The tPT was different from postoperative actual PT significantly (*p* < 0.001). The clinical evaluations were not correlated with postoperative PT.

**Conclusion:**

The abnormal PT is corrected by spinal osteotomy but is hard to restore to theoretical normal value. PT is a helpful parameter in making surgery plan. But pursuing postoperative PT being totally equal to tPT is undesirable and even may cause for overcorrection.

## Background

Ankylosing spondylitis (AS) is a chronic inflammatory rheumatic disease that leads to spinal kyphotic deformity in the late stage [1]. Patients with spinal kyphosis need to extend hip joints and rotate pelvis posterior to compensate for sagittal imbalance and get horizontal visual field. With development of the disease, spinal deformity exceeding the patient’s compensatory ability, severe AS patients will suffer from functional and structural impairments [1, 2]. Adopting spinal osteotomy to restore optimal sagittal balance is necessary for patients with AS spinal kyphosis and is critical to obtain satisfactory clinical results.

Various osteotomy technics have been described to restore sagittal balance for AS kyphotic deformity, among which pedicel subtraction osteotomy, vertebral column resection, and vertebral column decancellation can provide effective correction [3]. Despite of complex surgical techniques, surgery planning is also one of the formidable challenges. Van Royen et al. [4] developed a computer program to complete planning procedure. Suk et al. [5] suggested taking chin-brow vertical angle (CBVA) into consideration to maintain horizontal gaze. Song et al. [6] described an accurate and reliable method for calculating the exact angle required for spinal osteotomy. This method is an individualized plan, and the principle is to shift the gravity center of the trunk over the hip axis, which insures pelvic and lower extremity joints are in a neutral position postoperatively. Vialle et al. [7] reported a correlation between pelvic incidence (PI) and pelvic tilt (PT), PT = 0.37 × PI − 7. This equation is used for calculating postoperative PT individually and the exact angle required for spinal osteotomy in Song’s research [6]. They insist restoring PT to calculated value is most important in reconstructing sagittal balance.

In our clinical practice, however, we found that PT was hardly restored to calculated value in some of cases. These patients did not have ideal PT as calculated but still have satisfying outcome, whereas some of the patients who restored to calculated value was overcorrected and still did not have normal horizontal visual field after surgery. Therefore, the purposes of the present study were to describe the variables of PT before and after surgery and to investigate its impact on clinical outcomes.

## Methods

Twenty-three AS patients, including 2 women and 21 men, with thoracolumbar kyphosis who underwent spinal osteotomy in our center between June 2013 and March 2015 were studied. The operative strategy for every patient was planned individually according to Song’s method. The pre- and postoperative full-length spine radiographs of patients standing in neutral position, including the spine and pelvis, were obtained for all patients. The radiographic parameters included PT (angle between the vertical plane and a straight line joining the centers of the femoral heads and center of the sacral endplate), sacral slope (SS, angle between the sacral endplate and the horizontal plane), pelvic incidence (angle between a line drawn from center of hip axis to the center of the superior endplate of S1 and perpendicular to the endplate), and sagittal vertical axis (SVA, distance between the C7 plumb line and the superior posterior corner of S1). The osteotomy angle (OA) was also recorded. The theoretical pelvic tilt (tPT) was calculated by the formula tPT = 0.37 × PI − 7. All measurements were performed with dedicated software (Surgimap, New York, NY, USA). Clinical outcomes were performed with the Oswestry disability index (ODI) and Scoliosis Research Society-22 (SRS-22) surveys. The parameter measurement and clinical assessment were performed preoperative, postoperative immediately, and 2 years later after surgery (28 months on average), respectively. The measurements were made by a radiologist and a spine surgeon, and the average values were recorded. Surgical complications were also recorded.

The analyses were performed with the Statistical Package for the Social Sciences version 22.0 (SPSS Inc., Chicago, IL, USA). Differences between the preoperative and postoperative parameters were compared with independent samples’ *t* test. The relationship between postoperative PT and clinical outcome was evaluated by Pearson’s correlation coefficient. *P <* 0.05 was considered significant.

## Results

Mean age of the patients (2 women, 21 men) was 39.8 ± 9.1 years. Mean follow-up was 27.4 ± 3.8 months and at least 24 months (Table 1).

**Table 1:** Demographics of patients

Parameters	Mean ± SD	Range
Age(years)	39.8 ± 9.1	22–55
Gender	2 women, 21 men	
Follow-up (month)	27.4 ± 3.8	24–38

**Fig. 1:**
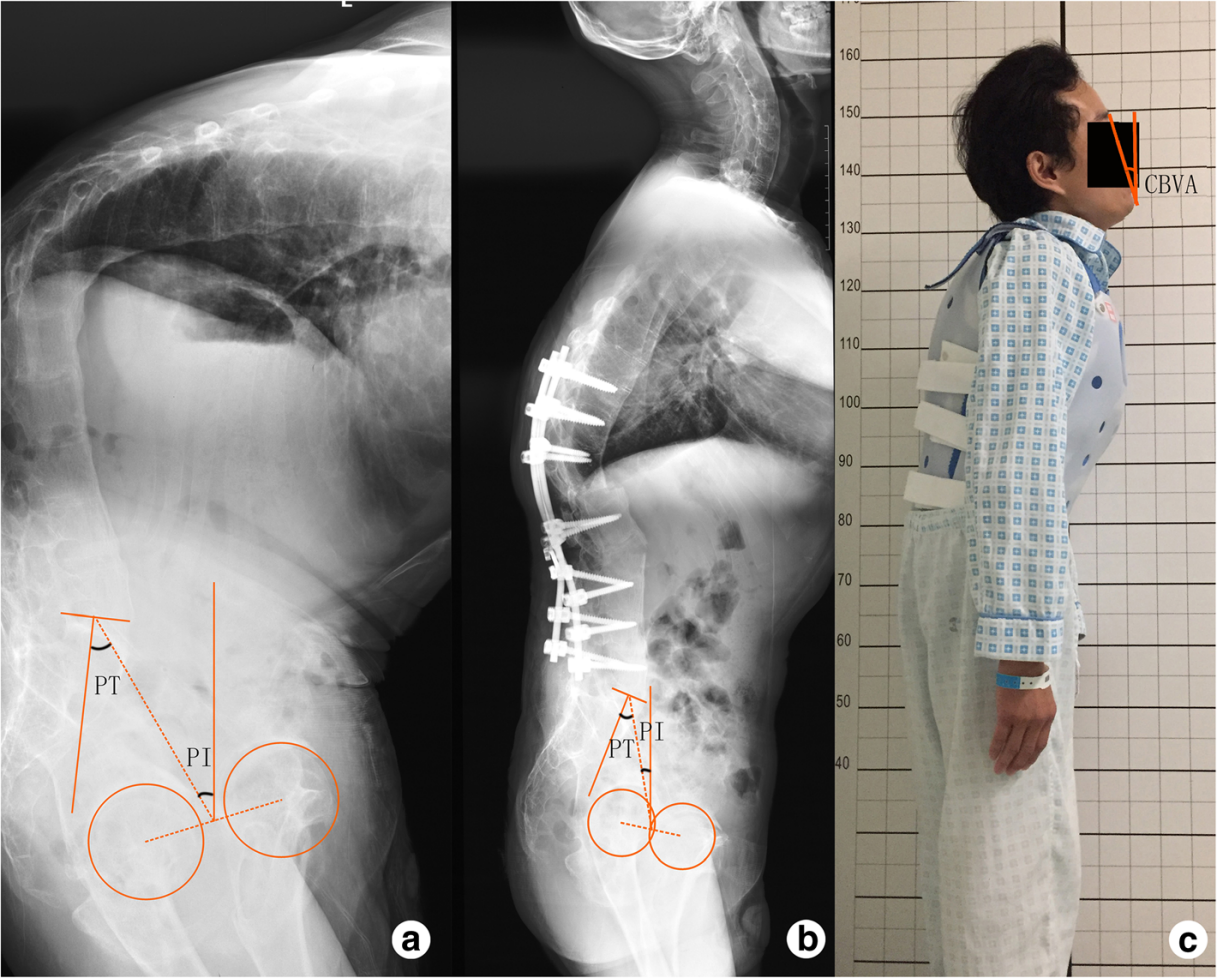
A 36-year-old men with ankylosing spondylitis with cervical ankylosis. **a** Preoperatively, PI and PT were 35° and 28°, respectively. tPT = 0.37 × 35° − 7° = 5.95°. **b** An interrupt-two-level osteotomy was adopted on T12 and L2, and the spine was fused from T9 to L5. PI and PT were 35° and 8°, respectively. **c** However, the postoperative CBVA was 16° upward and the patient was not able to look straight

The preoperative and postoperative radiographic evaluations of pelvic parameters are shown in Table 2. SS and SVA were corrected from 11.9° ± 11.2° and 18.0 ± 7.6 cm preoperatively to 25.8° ± 8.1° and 9.6 ± 6.3 cm postoperatively, respectively (*p* < 0.001). PT also experienced a sharp reduction from 37.6° ± 12.1° to 21.8° ± 9.8° postoperatively (*p* < 0.001). The tPT was calculated before surgery individually with mean value of 11.3° ± 3.6°. However, it was significantly different from postoperative actual PT (21.8° ± 9.8°, *p* < 0.001). The difference between tPT and postoperative PT of each patient was also calculated with the average of 10.9° ± 8.6°, range from − 3° to 25.2°, which was significantly different from the expected value, 0° (*p* < 0.001). This difference was within 5° in only 6 patients (26%), which can be considered as expected. In these 6 patients, there were 3 patients with cervical ankylosis and did not have a horizontal visual field after surgery (Fig. 1). In the rest 17 patients, there were 11 patients without cervical ankylosis having greater postoperative PT than tPT.

**Table 2:** Comparison of radiological parameters

Parameters	Preoperative (*n* = 23)	Postoperative (*n* = 23)	*p*
Sacral slop (°)	11.9 ± 11.2	25.8 ± 8.1	0.00*
Pelvic incidence (°)	49.4 ± 9.6	48.3 ± 7.4	0.65
Pelvic tilt (°)	37.6 ± 12.1	21.8 ± 9.8	0.00*
Sagittal vertical axis (cm)	18.0 ± 7.6	9.6 ± 6.3	0.00*

*Statistically significant if *p* < 0.05

The clinical evaluations at 2-year follow-up were significantly improved compared to preoperative findings (*p* < 0.001) (Table 3). But we did not observed a strong correlation between postoperative PT and clinical outcomes (SRS-22: *r* = 0.238, *p* = 0.275; ODI: *r* = − 0.155, *p* = 0.479).

**Table 3:** Comparison of health related quality of life

	Preoperative (*n* = 23)	2-year FU (*n* = 23)	*p*
SRS-22			
Appearance	1.6 ± 0.4(1.0–2.0)	4.5 ± 0.4(4.0–5.0)	0.00*
Mental	2.2 ± 0.9(1.0–4.0)	4.3 ± 0.5(3.0–5.0)	0.00*
Pain	2.3 ± 0.8(1.0–3.0)	4.5 ± 0.4(4.0–5.0)	0.00*
Function	2.6 ± 0.6(2.0–3.0)	4.4 ± 0.3(4.0–5.0)	0.00*
Satisfaction	1.5 ± 0.3(1.0–2.0)	4.8 ± 0.3(4.0–5.0)	0.00*
ODI			
Walking	0.42 ± 0.11(0.20–0.60)	0.07 ± 0.08(0–0.40)	0.00*
Sitting	0.33 ± 0.15(0.20–0.60)	0.05 ± 0.10(0–0.30)	0.00*
Standing	0.36 ± 0.07(0.20–0.80)	0.08 ± 0.06(0–0.40)	0.00*
Total	0.40 ± 0.11(0.22–0.55)	0.06 ± 0.07(0–0.22)	0.00*

*Statistically significant if *p* < 0.05

*SRS* Scoliosis Research Society, *ODI* Oswestry disability index

Surgical complications were observed after operation. There were 9 intraoperative dura tears. Abdominal muscles tense occurred in all the patients but recovered 2 weeks after operation. Five patients suffered from temporary lower extremities pain and weakness. Internal fixation failure and loss of correction were not found during follow-up.

## Discussion

As the course of the disease progress, the spinal kyphosis of the patients with AS is gradually aggravated and the gravity center moves anterior accordingly. To compensate for the sagittal imbalance, AS patients retrovert the pelvis and PT is found increasing [6, 8, 9]. In previous studies, PT was regarded as an essential parameter to analyze the sagittal alignment of AS patients with spinal kyphosis [6, 8, 10].

It has been widely recognized that establishing sagittal balance is the main purpose of spinal reconstruction operation [11]. The correction of sagittal imbalance is achieved through two aspects: reduction of spinal kyphosis and anterior rotation of pelvis. There were quite a lot of methods to help planning. Traditionally, a paper cutting is used for simulating the process of osteotomy and an exact OA is determined [12]. In order to achieve a better reconstruction of sagittal balance, Ondra et al. [11] used SVA = 0 cm as a balance standard. This method mainly concentrates on spinal sagittal alignment but ignores pelvic compensatory. As PT plays an important role in assessing sagittal and pelvic balance, it serves as one of substantial bases in later methods. Ruf et al. [13] suggested that the patient is able to adopt an ergonomic upright position when PT and gravity line are normalized. Debarge et al. [14] suggested that PT should be reduced to around 20° in order to regain pelvic orientation. Vialle et al. [7] studied 300 asymptomatic subjects and established a regression model of PT and PI: PT = 0.37 × PI − 7. Song et al. [6] defined postoperative PT according to this equation and calculated exact angle required for osteotomy. This method is individualized and takes spinal sequence and pelvic compensation together in to consideration.

We observed that the surgery significantly reduces SVA and PT and widens SS, while PI remains a constant value pre- and postoperatively. These findings are consistent with previous studies [7, 9, 14, 15]. However, we notice that PT was hardly restored to calculated value postoperatively as respected. We compared the actual postoperative PT and tPT of each patient, and significant difference was found to exist. There are two probable main reasons for this phenomenon. Firstly, the CBVA was taken into consideration when planning. Secondly, the OA during operation was not as accurate as calculated.

With the continuous aggravation of the spinal kyphosis, AS patients have to tilt the head backward to maintain horizontal visual field. Consequently, in most AS thoracolumbar kyphosis patients, the cervical spine usually fuses in an extended position. This becomes a tough problem of surgical strategy. Besides establishing sagittal balance, CBVA is very important in correction of thoracolumbar kyphotic deformity in AS, especially in patients with cervical ankylosis [6, 16]. Suk et al. [5] recommended that patients with CBVA between − 10° and 10° had better horizontal gaze and the patients were satisfied with their head-neck appearance. But another study reported that 10° < CBVA < 20° was the most suitable range that patients with ankylosed cervical spine had the better satisfaction in their daily life [16]. In the Song’s method, they also noticed the impact of CBVA and a smaller angle was considered better for osteotomy [6]. When CBVA is taken into consideration, a smaller OA is adopted. Consequently, the pelvis rotate less anteriorly, and the postoperative PT cannot be restored to tPT. If a planning was made without consideration of CBVA, the calculated OA will lead to excessive correction and the patient will look upward postoperatively.

The method we adopted can provide a personalized and accurate angle required for spinal osteotomy for each patient. But during the operation, to control the exact OA is very difficult. Based on our experience, every 1-cm osteotomy on lamina can bring 10° correction of kyphosis approximately. This is a simple but inaccurate method to control OA in surgery. In correction of spinal kyphosis, beside the operation of osteotomy, bending the rod and reduction of patient’s posture also influence the effect of correction to a large extent. In these processes, however, a precise OA is almost impossible to achieve and is usually less than the planned OA. As a consequence, the postoperative PT is hardly restored to tPT.

Nonetheless, although PT was not restored to tPT as planning, we did not observed a correlation between postoperative PT and clinical outcomes. Shin et al. studied 107 AS patients and suggested that SVA was a significant parameter in prediction of clinical outcomes but PT was not [17]. SVA is a parameter reflecting the balance of sagittal plane. The rotation of pelvis, reflected by PT, helps the recovery process of sagittal imbalance. However, there is not a strong correlation between PT and SVA in postoperative AS patients [15]. In calculating OA for AS patients, PT can be served as an important basis for reference. But it is unnecessary to pursue postoperative PT being equal to tPT excessively.

Major surgical complications recorded in this research are dura tears, abdominal muscles tense and temporary lower extremities pain and weakness. These do not influence sagittal balance. Internal fixation failure and loss of correction may have effect on PT, but these are not found during follow-up. A larger number of patients should be included in further research, and longer follow-up time should be studied.

There are also some limitations of this study. Firstly, the patient may not stand in a standard neutral position when taking X-rays. Therefore, some data may not be accurate enough due to the position. Secondly, if the radiographic parameters were measured twice or by a second researcher, a more credible data will be collected. These may lead to the existence of measurement bias. Thirdly, since PT is correlated with pelvic rotation, the influence caused by motion of hips and knees should be taken into consideration. Because of incomplete imaging data of lower extremities, this part of research has not been carried. But it is worth a further intensive study.

## Conclusions

The abnormal PT is observed decreasing after spinal osteotomy in AS thoracolumbar kyphosis patients. But in most of patients, PT cannot be restored to theoretical normal value. There are two main reasons. For one thing, a smaller OA have to be adopted in patients with cervical ankylosis. For another, although we have an accurate and individualized method to calculate the angle for spinal osteotomy, it is still hard to control to apply an exact OA during surgery. Nevertheless, the postoperative PT does not correlate with clinical outcome. PT is a helpful parameter in making surgery plan. But pursuing postoperative PT being totally equal to tPT is undesirable and even may cause for overcorrection.

## Abbreviations

AS: Ankylosing spondylitis
CBVA: Chin-brow vertical angle
OA: Osteotomy angle
ODI: Oswestry disability index
PI: Pelvic incidence
PT: Pelvic tilt
SRS-22: Scoliosis Research Society-22
SS: Sacral slope
SVA: Sagittal vertical axis
tPT: Theoretical pelvic tilt

## References

[1] Braun J, Sieper J (2007). Ankylosing spondylitis. Lancet.

[2] Sengupta R, Stone MA (2007). The assessment of ankylosing spondylitis in clinical practice. Nat Clin Pract Rheumatol.

[3] Zhao Y, Wang Y, Wang Z, Zhang X, Mao K, Zhang Y (2017). Effect and strategy of 1-stage interrupted 2-level transpedicular wedge osteotomy for correcting severe kyphotic deformities in ankylosing spondylitis. Clin Spine Surg.

[4] Van Royen BJ, De Gast A, Smit TH (2000). Deformity planning for sagittal plane corrective osteotomies of the spine in ankylosing spondylitis. Eur Spine J.

[5] Suk KS, Kim KT, Lee SH, Kim JM (2003). Significance of chin-brow vertical angle in correction of kyphotic deformity of ankylosing spondylitis patients. Spine (Phila Pa 1976).

[6] Song K, Zheng G, Zhang Y, Zhang X, Mao K, Wang Y (2013). A new method for calculating the exact angle required for spinal osteotomy. Spine (Phila Pa 1976).

[7] Vialle R, Levassor N, Rillardon L, Templier A, Skalli W, Guigui P (2005). Radiographic analysis of the sagittal alignment and balance of the spine in asymptomatic subjects. J Bone Joint Surg Am.

[8] Hu J, Ji ML, Qian BP (2014). Can pelvic tilt be predicated by the sacrofemoral-pubic angel in patients with thoracolumbar kyphosis secondary to ankylosing spondylitis. Spine (Phila Pa 1976).

[9] Hu J, Qian BP, Qiu Y, Wang B, Yu Y, Zhu ZZ (2017). Can acetabular orientation be restored by lumbar pedicle subtraction osteotomy in ankylosing spondylitis patients with thoracolumbar kyphosis. Eur Spine J.

[10] Duval-Beaupère G, Schmidt C, Cosson P (1992). A Barycentremetric study of the sagittal shape of spine and pelvis: the conditions required for an economic standing position. Ann Biomed Eng.

[11] Ondra SL, Marzouk S, Koski T, Silva F, Salehi S (2006). Mathematical calculation of pedicle subtraction osteotomy size to allow precision correction of fixed sagittal deformity. Spine (Phila Pa 1976).

[12] Van Royen BJ, De Gast A (1999). Lumbar osteotomy for correction of thoracolumbar kyphotic deformity in ankylosing spondylitis. A structured review of three methods of treatment. Ann Rheum Dis.

[13] Ruf M, Wagner R, Merk H (2006). Preoperative planning and computer assisted surgery in ankylosing spondylitis. Z Orthop Ihre Grenzgeb.

[14] Debarge R, Demey G, Roussouly P (2010). Radiological analysis of ankylosing spondylitis patients with severe kyphosis before and after pedicle subtraction osteotomy. Eur Spine J.

[15] Pan T, Qian BP, Qiu Y (2016). Comparison of sagittal spinopelvic alignment in patients with ankylosing spondylitis and thoracolumbar fracture. Medicine (Baltimore).

[16] Song K, Su X, Zhang Y (2016). Optimal chin-brow vertical angle for sagittal visual fields in ankylosing spondylitis kyphosis. Eur Spine J.

[17] Shin JK, Lee JS, Goh TS, Son SM (2014). Correlation between clinical outcome and spinopelvic parameters in ankylosing spondylitis. Eur Spine J.

